# Friction-Induced Near-Infrared Emission and Its Mechanism

**DOI:** 10.3390/ma17205119

**Published:** 2024-10-20

**Authors:** Shaodong Hu, Junhao Li, Xuefeng Xu

**Affiliations:** School of Technology, Beijing Forestry University, Beijing 100083, China; hushaodong@bjfu.edu.cn (S.H.); ljh229@bjfu.edu.cn (J.L.)

**Keywords:** triboluminescence, near-infrared light, electronic transition, energy level spacing, redshift

## Abstract

Triboluminescence (TL) is an optical phenomenon in which light is emitted from the surface of a material when subjected to pressure or shear forces. Due to its potential applications in non-destructive testing, radiation sources, and spectroscopic probes, TL has garnered increasing attention over the past two decades. However, experimental observations in the infrared spectrum remain limited, and its emission mechanism has not yet been fully understood. In this study, significant emission in the near-infrared spectrum was experimentally observed from the tribo-pairs of Cr/YSZ and quartz/YSZ. The results indicate that the Tribo-Induced Near-Infrared Light Emission consists of three peaks, in which the 780 nm peak is related to the electronic transition between the 3d_5/2_ and 3d_3/2_ orbitals of Y^3+^ ions, while the 880 nm and 990 nm peaks can be attributed to hole centers and T-type centers in the intrinsic defects of YSZ, respectively. Additionally, experiments reveal that the Cr/YSZ tribo-pair exhibits a redshift of 11–18 nm at the 780 nm peak compared to the quartz/YSZ tribo-pair. To explain the cause of the redshift phenomenon, X-ray photoelectron spectroscopy and UV-Vis absorption spectroscopy were used to measure the energy level spacing between the 3d_5/2_ and 3d_3/2_ orbitals of Y^3+^ and the bandgap width of YSZ before and after friction, respectively. We found that the bandgap width of the doped YSZ decreases after friction, which is often accompanied by a reduction in the energy level spacing between the 3d_5/2_ and 3d_3/2_ orbitals of Y^3+^. The extent of the reduction in energy level spacing varies with different dopants, leading to the redshift phenomenon.

## 1. Introduction

Triboluminescence (TL) refers to the light emitted by solid materials when subjected to stress or fracture [1,2,3,4,5]. Since the phenomenon was first recorded by Francis Bacon in 1605, it has garnered widespread attention from scholars both domestically and internationally. According to the relevant literature, approximately 36% of inorganic substances, 19% of organic compounds, 37% of aromatic compounds, 70% of alkaloids, and 50% of crystalline materials exhibit triboluminescent properties [6]. TL has broad applications in fields such as radiation sources [7,8], nondestructive testing [9,10], and display and lighting technologies [11,12]. However, due to its weak emission intensity, the quantitative detection of TL only became possible with the advent of photomultiplier tubes in the 1970s.

The earliest detected TL was in the visible spectrum. ZnS/Mn is one of the most extensively studied triboluminescent materials, with its emission spectrum ranging approximately from 550 to 650 nm [9,13]. In addition, significant visible spectrum emission has also been observed during sliding friction between tribo-pairs, such as SiO_2_/Al_2_O_3_ [14,15], Al_2_O_3_/ZnS [16], and MgAl_2_O_4_/ZnS [16]. In 2002, Japanese researcher K. Nakayama et al. [17] observed that the contact area emitted predominantly ultraviolet electromagnetic waves during the sliding friction between a diamond tip and a sapphire disk. Subsequently, Li Na et al. [18] discovered ultraviolet emissions ranging from 316 nm to 427 nm during friction between SiO_2_ and Al_2_O_3_. In 2009, J. Horvat et al. [19] observed terahertz radiation through the peeling of adhesive tapes. In the shorter wavelength X-ray range, J. R. Hird et al. [20] reported in 2011 that silicon resin and metal-loaded epoxy resin, when repeatedly contacted and separated in a vacuum environment, emitted X-rays with a photon rate of up to 10^8^ photons per second. In 2013, A. L. Collins et al. [21] observed X-rays with peak energies of up to 40 keV emitted from rolling friction between metals and insulators in a vacuum environment.

Experiments have also observed TL in the infrared spectrum [22,23,24,25]. Infrared light has broad applications in non-destructive testing [26], fiber optic communication [27], and medical treatments [28,29]. Therefore, triboluminescent infrared emission, as a potential infrared light source, holds significant importance. However, to date, there have been relatively few experimental observations of TL emitting infrared light, and its emission mechanism is not yet fully understood. Song et al. [22] found that metals such as molybdenum, chromium, iron, and aluminum emit infrared light during sliding friction with quartz. They proposed that metal doping on the surface of silicon dioxide during friction induces the formation of E’ centers and electron–hole pairs, which lead to electronic transitions and, thus, infrared light emission. Wang et al. [23] discovered that the carbon dioxide atmosphere can increase the intensity of near-infrared light emission from friction between silicon dioxide and Yttria-stabilized Zirconia (YSZ) by approximately 300 times, suggesting that this involves chemical reactions, impurities, and vacancies. Nakayama’s group [17] observed infrared photons during sliding contact between a diamond pin and a quartz disk, speculating that these photons originate from thermoluminescence of defects and impurity centers, blackbody radiation, and plasma luminescence. Additionally, Li et al. [24] found that pressure-induced lattice distortion and doping of transition metal ions lead to a redshift in infrared emission.

In this study, experiments revealed that TL from the tribo-pairs of chromium(Cr)/YSZ and quartz/YSZ results in near-infrared photon emission between 780 and 1000 nm. Compared to the quartz/YSZ tribo-pair, the Cr/YSZ tribo-pair exhibits a significant redshift at the 780 nm peak. Measurements using X-ray photoelectron spectroscopy (XPS) and ultraviolet–visible (UV-Vis) absorption spectroscopy indicate that the emission of near-infrared light in this wavelength range involves electronic orbital transitions, intrinsic defects, and impurity doping. The observed redshift is attributed to the narrowing of the material’s bandgap and the reduction in the energy level spacing of Y^3+^ ions due to doping.

## 2. Experimental Setup and Materials

**Figure 1:**
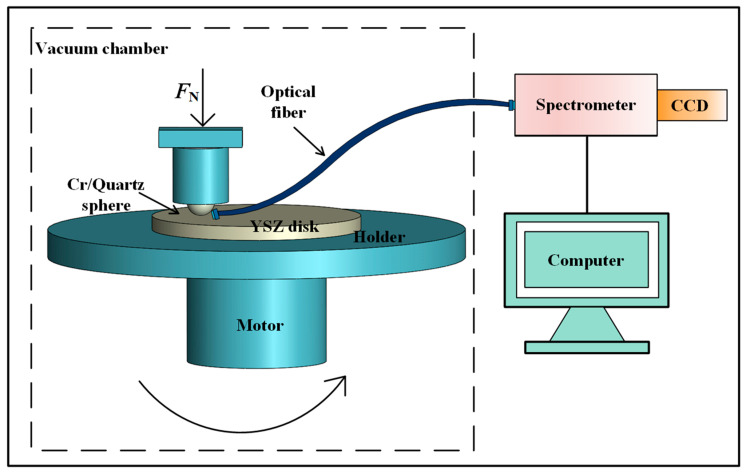
Experimental setup for measuring TL intensity and spectra.

The experimental setup is shown in Figure 1. The experiment uses a ball-on-disk sliding friction configuration. A stationary ball is pressed onto the surface of a rotating disk with a specified normal force while a motor drives the disk to achieve sliding friction between the tribo-pairs. The normal force *F*_N_ ranges from 0 to 10 N, and the motor speed *V* ranges from 0 to 3000 r/min. Photons generated in the contact area during friction are transmitted through optical fibers to a spectrometer (ANDOR, SR-500i-B1, Belfast, Northern Ireland, UK). The spectrometer is equipped with a deeply cooled, low-noise CCD camera (ANDOR, iDus 420 Series-BU2, Belfast, Northern Ireland, UK). The spectrometer’s measurement range is 200–1200 nm, with a wavelength resolution of 0.05 nm and an accuracy of ±0.2 nm.

YSZ is widely studied as a luminescent material due to its double-charged oxygen vacancies and three paramagnetic luminescent centers [30]. In the experiment, a YSZ disk with a diameter of 30 mm and a thickness of 2 mm was used as the lower tribo-pair, while the upper tribo-pair was a 4 mm diameter Cr ball or quartz ball. The purity of the Cr was 99.9%, and the purity of the quartz was 99.99%, both provided by the Shanghai Institute of Optics and Fine Mechanics, Chinese Academy of Sciences (Shanghai, China). During the experiment, TL intensity was measured under two conditions: first, by varying the relative sliding speed (100–600 r/min) with a fixed normal force (*F*_N_ = 5 N); second, by varying the normal force (1–6 N) with a fixed relative sliding speed (*V* = 500 r/min). Additionally, spectral measurements of both tribo-pairs were conducted in the wavelength range of 250–1200 nm under atmospheric conditions (with a humidity of 35 ± 0.5%) and vacuum conditions (with pressure between 1 and 10 Pa), with a normal force of *F*_N_ = 5 N and a speed of *V* = 500 r/min. The experimental temperature was between 22.5 °C and 23.5 °C. Finally, an X-ray photoelectron spectrometer (Thermo Fisher K-Alpha, Waltham, MA, USA) was used to measure the elemental composition of the lower disk before and after friction, as well as the XPS spectra of Y^3+^ in the 3d_5/2_ and 3d_3/2_ orbitals. The bandgap of YSZ before and after friction was measured using a UV-Vis absorption spectrometer (Shimadzu UV-2550, Kyoto, Japan).

## 3. Results and Discussion

### 3.1. Tribo-Emission of Near-Infrared Light

**Figure 2:**
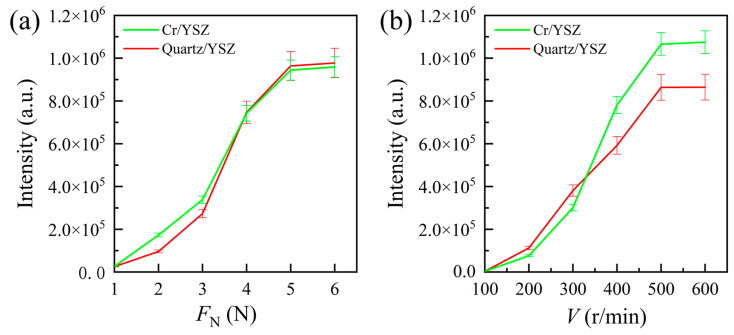
Images of photons emitted during the sliding contact between a Cr/quartz ball and a YSZ wafer in atmospheric conditions, with an integration time of 30 s: (**a**) *V* = 500 r/min, *F*_N_ = 1–6 N; (**b**) *F*_N_ = 5 N, *V* = 100–600 r/min.

The influence of rotational speed and normal load on tribo-induced light emission intensity in atmospheric conditions was first measured. Figure 2a shows the TL intensity during the sliding friction of Cr/YSZ and quartz/YSZ at a rotational speed of *V* = 500 r/min with normal loads of *F*_N_ = 1, 2, 3, 4, 5, and 6 N, while Figure 2b illustrates the TL intensity at a normal load of *F*_N_ = 5 N and rotational speeds of *V* = 100, 200, 300, 400, 500, and 600 r/min. The integration time for both experiments was 30 s. It can be observed that the emission intensity between the friction pairs increases with the rise in load and relative sliding speed. This is because, as the load and rotational speed increase, more mechanical energy is input during the friction process, leading to the generation of more photons. The experimental results also indicate that the interaction between TL intensity and load, as well as speed, is not a simple linear relationship. The quantitative relationship between TL intensity and load, as well as speed, will be studied in future work.

**Figure 3:**
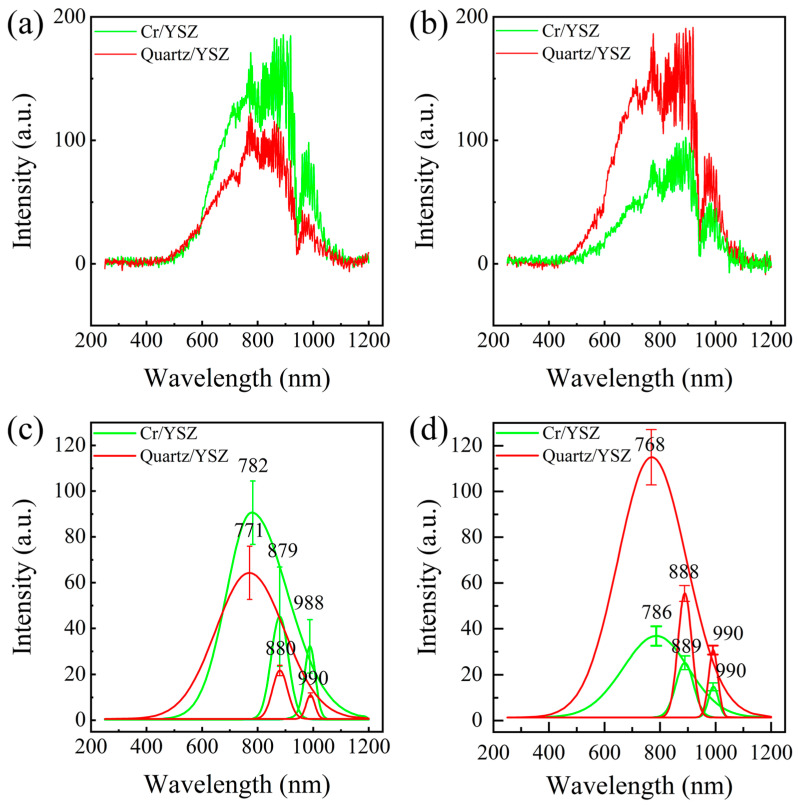
Spectra of photons emitted during the sliding contact between a Cr/quartz ball and a YSZ wafer under conditions of *F*_N_ = 5 N and *V* = 500 r/min, with an integration time of 240 s: (**a**) luminescence spectrum in an atmospheric environment; (**b**) luminescence spectrum in a vacuum environment; (**c**) Gaussian peak fitting spectrum in an atmospheric environment; (**d**) Gaussian peak fitting spectrum in a vacuum environment.

To better characterize the TL phenomenon, the emission spectra of both tribo-pairs were examined under conditions of *F*_N_ = 5 N and *V* = 500 r/min, with a total measurement time of 240 s. The TL spectra of the Cr/YSZ and quartz/YSZ tribo-pairs in both atmospheric and vacuum environments, after being denoised using the Savitzky–Golay filter, which effectively removes noise while preserving local features of the data, are shown in Figure 3. The results indicate that in both environments, significant emission occurs in the near-infrared range (780–1000 nm) for both tribo-pairs. Through Gaussian peak fitting [31], three distinct peaks were identified at approximately 780 nm, 880 nm, and 990 nm, as shown in Figure 3c,d. In the atmospheric environment, the emission intensity of the Cr/YSZ tribo-pair was higher than that of the quartz/YSZ tribo-pair. However, in the vacuum environment, the quartz/YSZ tribo-pair exhibited significantly higher emission intensity compared to the Cr/YSZ tribo-pair. We speculate that the higher emission intensity of the Cr/YSZ tribo-pair in the atmospheric environment may be due to the oxidation of Cr with oxygen in the air, resulting in the formation of chromium oxide. The energy released during this oxidation process likely excites electron transitions within the material, leading to photon emission. In contrast, quartz, being a stable oxide, does not undergo significant oxidation during friction. The reason for the higher luminescence intensity of the quartz/YSZ tribo-pair in a vacuum environment compared to that of the Cr/YSZ tribo-pair may be due to the fact that quartz is a dielectric material, which facilitates charge separation during friction. At the same time, in a vacuum environment, the absence of air or other media reduces electron scattering, allowing more charges to recombine and emit photons. In contrast, due to the higher conductivity of metallic Cr, the charges generated during friction are dissipated more quickly within the metal rather than accumulating on the surface. As a result, the effect of charge separation is weaker, leading to a relatively lower photon emission. Additionally, it was observed that the Cr/YSZ tribo-pair consistently exhibited a redshift at the 780 nm peak compared to the quartz/YSZ tribo-pair, regardless of the environment.

### 3.2. TL Mechanism

**Figure 4:**
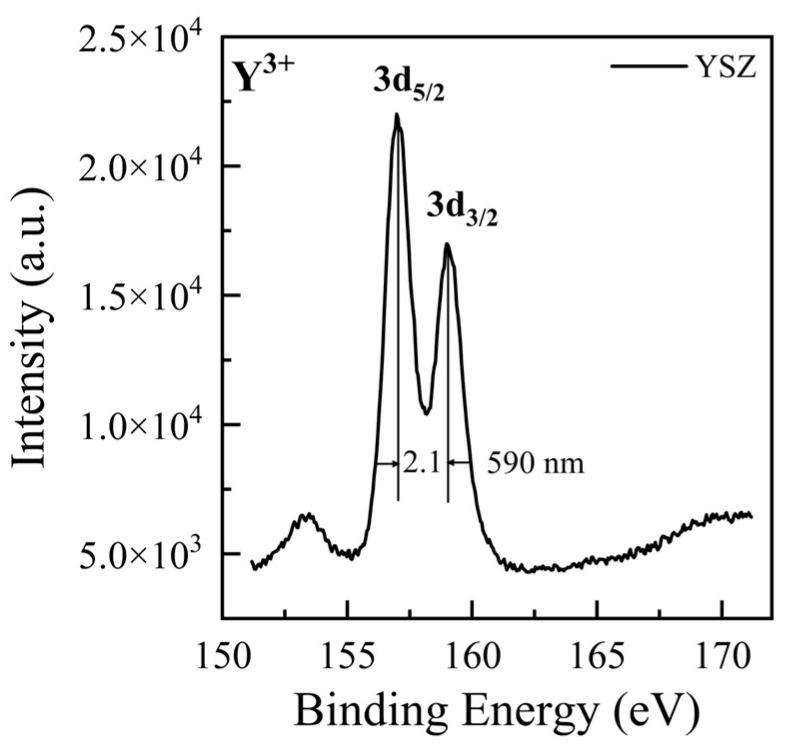
The XPS spectra of Y^3^⁺ ions at the 3d_5/2_ and 3d_3/2_ peaks on the YSZ surface, along with the energy level spacing between the peaks and the corresponding wavelengths.

During friction, the triboelectric effect generates an electric field, which can lead to the discharge of gas in the contact gap, resulting in light emission. These gas discharge peaks are typically narrow and mostly occur in the ultraviolet and visible wavelength regions [18,32], which do not match the shape and location of the emission peaks observed in this study. Additionally, considering that significant emission was observed in both vacuum and atmospheric conditions (as shown in Figure 3), the TL in this study is unlikely to be caused by the discharge of gas. Since the number and positions of emission peaks in the friction spectra of Cr/YSZ and quartz/YSZ are nearly identical, the source of the emission is likely from the YSZ on the lower tribo-pair. Previous studies on XPS measurements of YSZ have shown that electronic transitions between the 3d_5/2_ and 3d_3/2_ orbitals of Y^3+^ ions in YSZ result in the emission of photons near 708 nm [33,34]. Our XPS measurements show that Y^3^⁺ ions emit photons around 600 nm during this transition (as shown in Figure 4). Here, we attribute the emission peak at 780 nm to electronic transitions between the 3d_5/2_ and 3d_3/2_ orbitals of Y^3^⁺ ions in YSZ. The discrepancy between the photon wavelengths observed in the experiment and the XPS electronic structure measurements may be attributed to changes in the electronic structure caused by surface deformation during the friction process [35]. EPR measurements of YSZ show three stable paramagnetic luminescence centers at room temperature: an F^+^-type center (single ionized oxygen vacancy), a T-center (Zr^3^⁺ in a trigonal oxygen environment), and a hole center [30]. The peak near 880 nm is attributed to hole centers in YSZ [23], while the peak at 990 nm is related to T-centers in YSZ [23,36].

### 3.3. Mechanism of Redshift in TL

**Figure 5:**
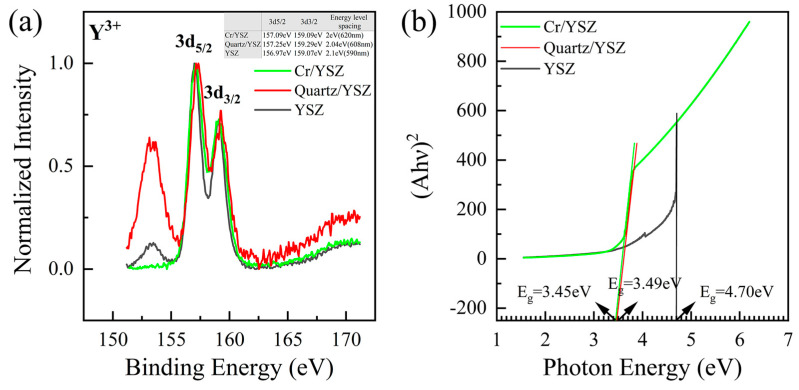
(**a**) The XPS spectra of Y^3^⁺ ions at the 3d_5/2_ and 3d_3/2_ peaks on the YSZ surface before and after friction for the tribo-pairs of Cr/YSZ and quartz/YSZ, along with the energy level spacing between the peaks, (**b**) optical absorption spectra drawn as (Ahν)^2^ vs. photon energy for Cr/YSZ and quartz/YSZ before and after friction. Extrapolation of the linear part of each graph gives the bandgap of the samples.

By comparing the TL spectra of the two tribo-pairs, it is observed that the emission peak of Cr/YSZ shows a significant redshift at 780 nm compared to that of quartz/YSZ, with a redshift distance of approximately 11–18 nm (as shown in Figure 3c,d). Since the emission peak at 780 nm originates from the electronic transition of Y^3+^ ions between the 3d_5/2_ and 3d_3/2_ orbitals, it is necessary to calculate the energy level spacing of Y^3+^ ions in YSZ. Based on the analysis of XPS spectra before and after friction (Figure 5a), we found that the energy level spacing of Y^3+^ ions on the YSZ surface decreased after friction for both tribo-pairs: the energy level spacing before friction was 2.1 eV, while after friction, it reduced to 2 eV for Cr/YSZ and 2.04 eV for quartz/YSZ. This reduction in energy level spacing causes the transition between the two energy levels to emit photons with longer wavelengths, resulting in a redshift. Since the energy level spacing on the YSZ surface decreases after friction for both the Cr/YSZ and quartz/YSZ pairs compared to before friction, both sliding pairs exhibit redshift phenomena. However, the energy level spacing between Cr/YSZ and quartz/YSZ after friction differs by 0.04 eV, which corresponds to a wavelength difference of 12 nm, consistent with the 12 nm redshift observed in the experimental spectra.

According to previous theoretical studies, changes in the energy level spacing are often accompanied by changes in the bandgap width [37]. To further explain the occurrence of the redshift phenomenon, we performed UV-Vis absorption spectroscopy on YSZ before and after friction to obtain its bandgap width, as shown in Figure 5b. Based on the formula proposed by Tauc, Davis, and Mott [38,39], we plotted (Ahν)^2^ as a function of photon energy and extrapolated the linear portion of the curve to the x-axis to determine the bandgap of the samples. We calculated the bandgap of un-doped YSZ to be 4.7 eV, which is slightly lower than the values reported in the literature [40]. In contrast, the bandgap of YSZ doped with Cr^3+^ and Si^4+^ was found to be 3.45 eV and 3.49 eV, respectively. It is evident that the bandgap width of doped YSZ significantly decreased, with the bandgap of Cr^3+^-doped YSZ being 0.04 eV narrower than that of Si^4+^-doped YSZ, a result that is consistent with the XPS measurements.

We hypothesize that the change in the energy level spacing of Y^3^⁺ may result from the doping of counter-surface elements during the friction process. To verify this, XPS was used to measure the elemental composition of the YSZ surface before and after friction. The results indicate that the elemental composition of the YSZ surface before and after the sliding experiment has changed. The elemental composition of YSZ before the sliding experiment has atomic percentages (atom %) of Zr = 34.55%, Y = 8.55%, C = 17.48%, and O = 39.42%. After sliding friction with Cr, the atomic composition of YSZ is Zr = 30.95%, Y = 7.31%, C = 14.38%, O = 38.24%, and Cr = 9.12%. After sliding friction with quartz, the atomic composition of YSZ is Zr = 13.38%, Y = 5.42%, C = 52.26%, O = 26.68%, and Si = 2.26%. Therefore, based on the differences in atomic composition before and after friction, we conclude that Cr and Si are implanted into the YSZ surface during the sliding contact with the respective materials. Moreover, the previous literature has reported that modifying the host crystal can lead to a shift in luminescence wavelength. This occurs because such doping not only creates new occupiable quantum states below the conduction band of the luminescent material but also reduces the energy level spacing and narrows the bandgap [41]. In this study, doping with Cr^3+^ and Si^4+^ resulted in a reduced bandgap, accompanied by a decrease in the energy level spacing between the 3d_5/2_ and 3d_3/2_ orbitals of Y^3+^ ions. As a result, the energy released by electrons during the transition between the two energy levels decreases, which is reflected in the spectrum as a redshift.

## 4. Conclusions

In the TL experiments involving metal Cr/YSZ and quartz/YSZ, three distinct emission peaks at 780 nm, 880 nm, and 990 nm in the near-infrared region were observed through ball-on-disk sliding friction. Additionally, a redshift of 11–18 nm was detected at the first peak (780 nm) for the Cr/YSZ tribo-pair compared to the quartz/YSZ tribo-pair. The formation of the 780 nm peak is attributed to the electronic transition of Y^3+^ ions between the 3d_5/2_ and 3d_3/2_ orbitals in YSZ, while the 880 nm and 990 nm peaks are related to the hole center and T-center within the intrinsic defects of YSZ, respectively. To investigate the cause of the redshift, XPS analysis was performed on Y^3+^ ions in YSZ after both friction experiments. The results revealed that, compared to the pre-friction state, the energy level spacing between the 3d_5/2_ and 3d_3/2_ orbitals of Y^3+^ in YSZ had decreased for both the Cr/YSZ and quartz/YSZ tribo-pairs leading to a redshift in both cases. However, there was a 0.04 eV difference in energy level spacing between the Cr/YSZ and quartz/YSZ tribo-pairs, corresponding to a wavelength shift of 12 nm, which aligns with the experimental findings. Additionally, UV-Vis absorption spectroscopy was conducted on YSZ before and after doping with Cr^3+^ and Si^4+^. The results showed that doping reduced the bandgap of YSZ, with Cr^3+^-doped YSZ exhibiting a 0.04 eV narrower bandgap compared to Si^4+^-doped YSZ, consistent with the XPS results. We hypothesize that the reduction in Y^3+^ energy level spacing arises from the incorporation of Cr^3+^ and Si^4+^ into the YSZ surface during friction. XPS analysis confirmed the doping of Cr^3+^ and Si^4+^ during the friction process. Based on the previous literature, we conclude that doping with Cr^3+^ and Si^4+^ narrows the bandgap of YSZ and reduces the energy spacing between the 3d_5/2_ and 3d_3/2_ orbitals of Y^3+^, thereby decreasing the energy released during electronic transitions and causing the observed redshift at the 780 nm peak.

## Data Availability

The original contributions presented in the study are included in the article. Further inquiries may be directed to the corresponding author.
